# Physical and Mechanical Evaluation of Silicone-Based Double-Layer Adhesive Patch Intended for Keloids and Scar Treatment Therapy

**DOI:** 10.3390/polym8110398

**Published:** 2016-11-22

**Authors:** Barbara Mikolaszek, Marzena Jamrógiewicz, Krystyna Mojsiewicz-Pieńkowska, Maria Żebrowska, Małgorzata Sznitowska, Justyna Strankowska

**Affiliations:** 1 Department of Pharmaceutical Technology, Faculty of Pharmacy with Subfaculty of Laboratory Medicine, Medical University of Gdansk, Al. Gen. Hallera 107, 80-416 Gdańsk, Poland; bmiko@gumed.edu.pl (B.M.); mariazebrowska@interia.eu (M.Ż.); msznito@gumed.edu.pl (M.S.); 2 Department of Physical Chemistry, Faculty of Pharmacy with Subfaculty of Laboratory Medicine, Medical University of Gdansk, Al. Gen. Hallera 107, 80-416 Gdańsk, Poland; kpienk@gumed.edu.pl; 3 Institute of Experimental Physics, Faculty of Mathematics, Physics and Informatics, University of Gdansk, Wita Stwosza 57, 80-308 Gdańsk, Poland; j.strankowska@ug.edu.pl

**Keywords:** adhesive silicone skin dressing, scar treatment, mechanical properties, tensile strength, morphology

## Abstract

Growing interest in silicone elastomers for pharmaceutical purposes is due to both their beneficial material effect for scar treatment and their potential as drug carriers. Regarding their morphological structure, silicone polymers possess unique properties, which enable a wide range of applicability possibilities. The present study focused on developing a double-layer adhesive silicone film (DLASil) by evaluating its physical and mechanical properties, morphology, and stability. DLASil suitability for treatment of scars and keloids was evaluated by measurement of tensile strength, elasticity modulus, and elongation. The results indicated that mechanical and physical properties of the developed product were satisfying.

## 1. Introduction

Usage of silicone polymers for pharmaceutical purposes has dynamically increased over the last few years. Both medical devices and dosage forms are being developed. Silicone materials can be found as excipients mostly in preparations for external use, such as gels, creams, patches, films, or dressings [1]. The possibility of incorporating pharmaceutically active compounds into the polymer network has been also explored. Nevertheless, for treatment of scars and keloids the most popular approach, also indicated in clinical recommendations [2,3], is the use of a silicone patch without active substances, since its beneficial influence on scars results from the material’s unique properties [4,5,6]. Polysiloxanes are formed into films, which are intended for application to the abnormally changed skin areas. Lack of a sensitising effect after extended skin contact and patch flexibility, which allows adaptation to the individual shape, are important features that enable long-term therapy resulting in softening and smoothing of scars [5,7,8]. Consequently, proper physical and mechanical parameters of the material are crucial to achieve the therapeutic effect.

There is a strong correlation between the polymer structure and physical as well as mechanical properties of the film. Molecular weight of the polymer, shape of the molecule, and intermolecular forces influence the molecular mobility, such as rotation or partial deformations. Silicones are significantly different from other polymers due to their weak attractive intermolecular forces. This is primarily due to a number of structural differences. Angles of the Si–O–Si bonds are wider than the angles of the C–O–C bonds; moreover, the Si–O bond is longer than the C–O–C and C–C bonds. Also, Si–O bond rotation is preferable to rotation of the C–C bond, and simultaneously free polymer chains block free rotation of methyl groups. As a result, materials whose physical properties can be modified are obtained, which is advantageous for pharmaceutical formulation and further applicability adjustment [9].

Medical devices indicated for prolonged implantation or skin contacts have to meet additional requirements (ISO 10993) [10]. Biocompatibility and skin nonsensitisation of silicone material are crucial properties; simultaneously, sufficient oxygen permeation with proper skin hydration are essential for maintenance of skin function and minimising side effects [11,12]. Mechanical properties in terms of patch applicability decide whether clinical usage of the silicone materials is possible [13]. Generally, they should be sufficiently resistant to stretching or permanent deformation, as well as flexible and soft enough to ensure the patient’s comfort when applied to the skin. Since therapy of scars requires prolonged patch application, mechanical properties of the film are expected to be maintained not only during its shelf life, but also for a whole period of therapy, with a single patch used for up to a few weeks, which can be regarded as stress conditions. Stability of the polymer matrix may be expressed by measurements of the flexibility, elasticity, and elongation [14]. In the previous work [15], in vitro characteristics of occlusiveness and permeability of oxygen and water vapour and in vivo assessment of skin hydration and adhesion for the novel silicone patch were presented.

The aim of this study was to evaluate the physical and mechanical properties of the adhesive silicone film DLASil (double-layer adhesive silicone film), including its stability under simulated stress conditions. A practical aspect of the work was to explore the possibility of adjusting mechanical behaviour in order to achieve successful treatment of scars or keloids. Adhesive films must adhere firmly to the scar surface, which is stiff and either sunken or raised above surrounding skin. Sufficient elasticity and softness of applied dressing is crucial to assure constant contact with the abnormal surface of the skin. Based on the review of literature, it can be concluded that no standard methodology for assessment of the properties of the silicone film, intended for skin application in form of a patch, has been so far introduced. A systematic approach would be useful in the development of medical materials intended for skin application. The measured macroscopic properties result from the chemical composition and structural construction of silicone film itself, but also significantly influence its applicability.

## 2. Experimental Section

A two-part platinum-catalysed silicone elastomer, Gumosil AD-1 (polydimethylsiloxane (PDMS)), was purchased from Silikony Polskie (Nowa Sarzyna, Poland), Soft Skin Adhesives DC 7-9850 Parts A & B and polydimethylsiloxane fluid Q7-9120 (silicone oil, SO) with kinematic viscosity of 350 cSt were obtained from Dow Corning (Wiesbaden, Germany). Commercially available silicone patches Codosil™ Adhesive (TZMO SA, Torun, Poland) and Cica-Care (Smith & Nephew, Hull, UK) were used for comparative purposes.

### 2.1. Preparation of Silicone Films

Gumosil AD-1 (PDMS) blends with silicone oil (SO) were prepared. Gumosil AD-1 Parts A & B mixing ratio was 9:1, by weight. The percentage of SO varied from 0% to 25% *w*/*w*. The preparation procedure started with mixing Part A of Gumosil AD-1 with SO, using a mechanical stirrer at 300 rpm (IKA RW 20, Labortechnik GmbH, Munich, Germany). Then, Part B of Gumosil AD-1 was added, followed by de-airing of the mixture in high vacuum. The nonadhesive layer (matrix) was formed by casting the resulting mass into a film of 500 μm thick. The curing process was conducted at 25 ± 1 °C for 24 h. Adhesive layer (DC) was obtained by mixing equal parts (1:1 ratio, *w*/*w*) of two-part silicone rubber (7-9850 Parts A & B). Nonadhesive matrices were coated with this DC blend to a thickness of 500–550 μm and cured at 25 ± 1 °C for 24 h. DLASil films were stored at room temperature in a controlled environment (25 ± 1 °C, RH 60%) for 7 days before analysis.

### 2.2. Morphology

Microscopic images were obtained using an optical microscope, Opta-Tech X2000, equipped with a 9 MP Opta-Tech CMOS digital camera with Opta View software for data acquisition, version 7.1.0.4 (Opta-Tech, Warsaw, Poland). The structure of DLASil was also visualised by fluorescence microscopy (Nikon, Eclipse 50i, Nikon Corporation, Tokyo, Japan) and examined using a set of filters (excitation filter, 510–560 nm; emission filter, 505 nm). Sodium fluorescein (Aldrich, Poznan, Poland) solution (1 mg/mL) in diethylene glycol was added to Part A of Gumosil before curing to observe its distribution in the nonadhesive matrix layer and to enable further observation of the contact border between layers. In case of the Cica-Care patch, a piece of the patch was soaked in the fluorescein for 3 days and then dried with a cotton cloth.

The morphology of DLASil was also examined by means of a scanning electron microscope (SEM, Hitachi TM-1000, Tokyo, Japan), with the accelerating voltage of 15 kV.

### 2.3. Mechanical Characteristics of Silicone Films

Mechanical properties were tested using a texture analyser (TA.XT plus, Stable Micro Systems, Godalming, UK). Hardness of the formulated films was determined using 45° acrylic conical probe in compression mode (test speed 1 mm/s, compression force of 5.0 g). Tensile grips in tension mode were used and set to maximum force (break point)—test speed of 1 mm/s, loading cell of 5 kg. Strips of silicone films 30 mm in length, with a cross-section area of 2.30 ± 0.40 mm^2^, were examined. For both tests, at least eight samples of each formulation were tested. Measurements were performed at 24 ± 1 °C and relative humidity of 35% ± 5%.

In order to describe elasticity and durability of the films, tensile strength, percentage of elongation at break (EB), and Young’s modulus were calculated from the obtained strain–stress curves [16].

Statistical analysis of the data was performed using a parametric one-way ANOVA with post hoc tests (Statistica 10.0, StatSoft, Cracow, Poland). In all cases, *p* < 0.05 denoted significance.

### 2.4. Adhesiveness of Double-Layer Adhesive Silicone Films

A probe tack test was performed to assess adhesiveness of the patch. Texture analyser with *a* spherical φ 5 mm stainless steel probe was used for the experiment (TA.XT plus, Stable Micro Systems, Godalming, UK). Test parameters were set to a contact time of 2 s and applied force of 2 N, with a test speed of 0.05 mm/s. The maximum force required to detach the probe from examined patches was considered as the adhesion force, and work of adhesion was calculated as the area under curve for Force = *f* (distance).

### 2.5. Stability Testing of DLASil

Tests were performed for the double-layer silicone dressing (DLASil) and commercially available silicone patches: Codosil™ and Cica-Care. The products were exposed to accelerated storage conditions for 28 days: in a climatic chamber (Binder, Tuttlingen, Germany), at temperature 40 °C and relative humidity 75%, in a refrigerator (4–6 °C, relative humidity 60%–70%), and in ambient conditions (25 °C, relative humidity 60%). Samples were also exposed to UVB irradiation in a Suntest CPS+ Atlas chamber (Accelerated Tabletop Exposure Systems, Atlas, Gelnhausen, Germany), equipped with Xenon lamp (1.1 to 1.5 kW) and two filters: special window glass and Solar ID65, applied together. The luminance was set at an exposure power of 500 W/m^2^ for 48 h. As a simulation of the maintenance of the patches by a patient during standard usage, the samples (8 cm^2^) were soaked in 2 mL of 2% aqueous solution of a hypoallergic liquid soap (Pollena, Poland) for 1 min, rinsed with 50 mL of water, and left to dry. The procedure was repeated 10 times for each sample.

### 2.6. Spectroscopic Analysis by ATR-FTIR

Silicone components and DLASil were studied by attenuated total reflectance–Fourier transform infrared (ATR–FTIR) spectroscopy. Spectra were registered by means of an FTIR-4100 spectrophotometer (Jasco, Cracow, Poland) and collected in the range 4000–650 cm^−1^ with 32 scans. ATR–FTIR spectra of DLASil elastomers placed directly on ZnSe crystal as the internal reflecting element (IRE) were obtained with the horizontal attenuated total reflectance (HATR) accessory (reflectance technique). Acquired spectra were first corrected for background, using the signal from pure crystal as a standard. DLASil and all silicone components were studied by ATR–FTIR before and after storage.

## 3. Results and Discussion

The silicone patch was prepared as a double-layer film, with an outer nonadhesive layer. The nonadhesive layer acts as a backing layer and prevents sticking of the patch to the clothing while applied to the skin. The inner adhesive layer must assure stable contact between the patch and the skin in order to hold the dressing in place. To ensure the comfort of the patient, both layers as a whole must be flexible and soft to eliminate discomfort and the possibility of adhesion failure during application due to muscles and joints movements.

**Figure 1:**
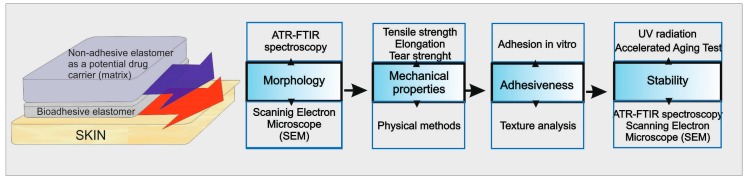
Algorithm of the procedure applied for the physical and mechanical characterisation and stability assessment of the adhesive silicone film double-layer adhesive silicone film (DLASil).

Figure 1 presents a set of the experiments performed during the study. The graph shows the physical structure of DLASil and parameters that were determined in order to assess applicability and stability of DLASil as dermal patches. It is proposed as a general strategy to characterise the adhesive silicone films as the development stage of dermal patches.

### 3.1. Silicone Films Morphology

Structure examination was performed using images obtained by optical and scanning electron microscopes. The adhesive layer and nonadhesive layer (with or without silicone oil), separately or combined into a double-layer patch, were examined.

**Figure 2:**
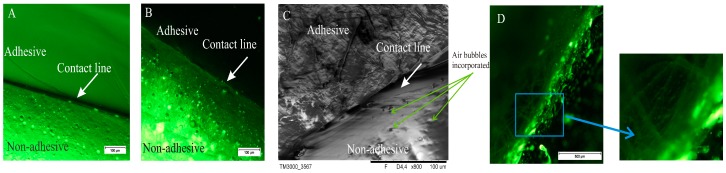
Images from a fluorescence microscope (**A**,**B**,**D**) and from an SEM (**C**) of DLASil (**A**,**C**); DLASil with silicone oil (SO) (**B**); and Cica-Care (**D**).

Figure 2 shows microscopic image of DLASil with the border line between both layers. In the two first images (Figure 2A,B) the contact line is straight and condensed, while the third image (Figure 2C), obtained by SEM, provides some details confirming an irregular and mobile structure of the silicone polymer. The matrix layer, consisting of nonadhesive elastomer, forms a thick structure, and air bubbles are visible. It should be emphasised that great attention must be paid during the mixing of the two components of Gumosil elastomer because of a tendency to entrap the air bubbles in a crosslinking structure. The adhesive layer, which directly contacts the skin, is characterised in the optical image (Figure 2A) by a very smooth and homogeneous structure, while the SEM image shows an irregular structure, resembling a jelly (Figure 2C). The silicone oil incorporated into the nonadhesive layer is visible as the lighter dots in the image (Figure 2B). In the case of the Cica-Care patch, its diverse structure on the border of the layers and some “thread” structures are observed (Figure 2D).

**Figure 3:**
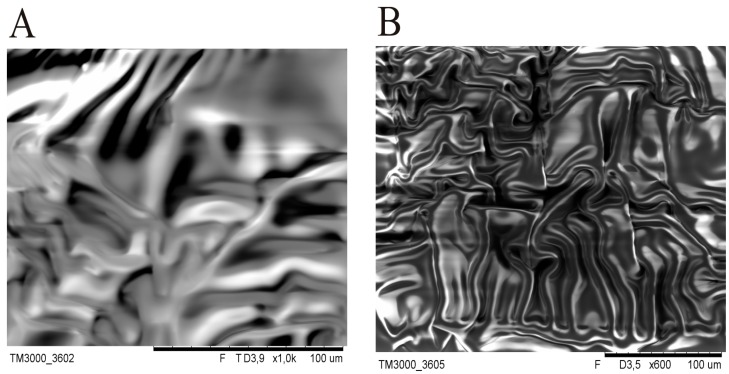
Deformation and flowing of the adhesive layer as a result of the electron bombardment in SEM: (**A**) 3.9 kV; (**B**) 3.5 kV.

At the early stage of the experiment, SEM images of silicone patches without gold sputtering of the samples were recorded. However, during SEM testing with electron bombardment, a significant difference in DLASil’s behaviour was noticed. The adhesive layer is very sensitive (Figure 3), while the nonadhesive layer is more resistant to high electrical potentials.

**Figure 4:**
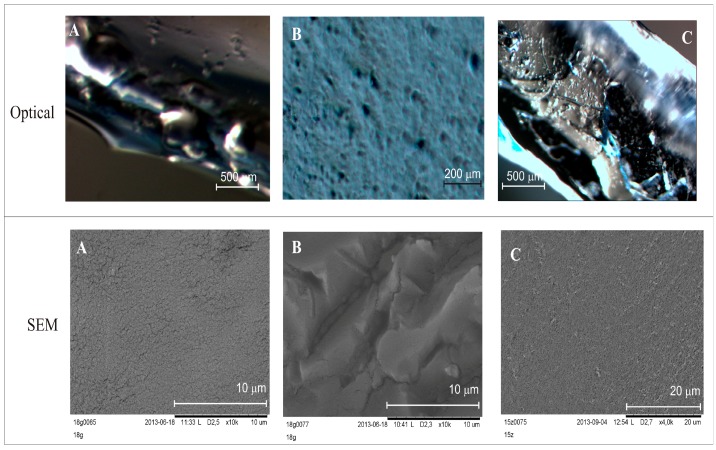
Images obtained by means of optical microscope and SEM: (**A**) adhesive silicone layer of DLASil; (**B**) nonadhesive silicone layer of DLASil; (**C**) Cica-Care patch.

The differences in the layers are clearly noticeable in the images of DLASil and in commercially available patches (Figure 4). Images obtained by the optical microscope correspond to the organoleptic appearance of silicone elastomers. A loose, gel-like structure of tacky, adhesive layer of DLASil and Cica-Care (Figure 4A,C) can be identified without difficulties in the microscopic images. This layer is homogenous. It provides the effective cohesion with the non-adhesive layer (Figure 4B).

In the SEM image (Figure 4B), the tight structure of matrix-reservoir can be noticed. In comparison to adhesive layer this layer is irregular and rigid. Silicone film DLASil, compared with other commercially available preparation (e.g., Cica-Care), possesses a rougher structure (Figure 4C). The adhesive surface of Cica-Care is considered the smoothest, which may partially explain that the lowest adhesion force values were observed for this product [15].

DLASil silicone film consists of two types of elastomers with a three-dimensional network structure. The structure is formed during the crosslinking by addition reaction, which occurs between two reactive silicone polymers containing different functional groups (our publications, 2011, 2015). Components of Gumosil Part A and DC Part B contain a polydimethylsiloxane (PDMS) with a SiH siloxane crosslinker, while Gumosil Part B and DC Part A carry vinyl-enblocked polymer with a reactive dimethylvinyl CH_2_=CH_2_ group with a platinum catalyst (Pt-Karstedt). The addition reaction (hydrosilylation) is initiated by mixing Parts A and B. In the process of crosslinking, binding of linear polymer molecules into spatial macromolecule occurs, resulting in so-called “bridges” being formed with no byproducts. In the course of the reaction, disruption of the multiple C=C bonds of the vinyl group occurs, and a Si–C bond is formed as a result. Consequently, multiple addition reaction of Si–H with vinyl groups form numerous bridges connecting hydrocarbon chains of polysiloxane. In the previous work [17], the molecular weights of the individual components, used to prepare the silicone elastomer, were determined. The increase in molecular weight of the polymer causes a change in its macroscopic properties, especially in physical properties of the material (e.g., increase of the melting point, strength, elasticity, and hardness).

### 3.2. Mechanical Characteristics of Silicone Patches

**Figure 5:**
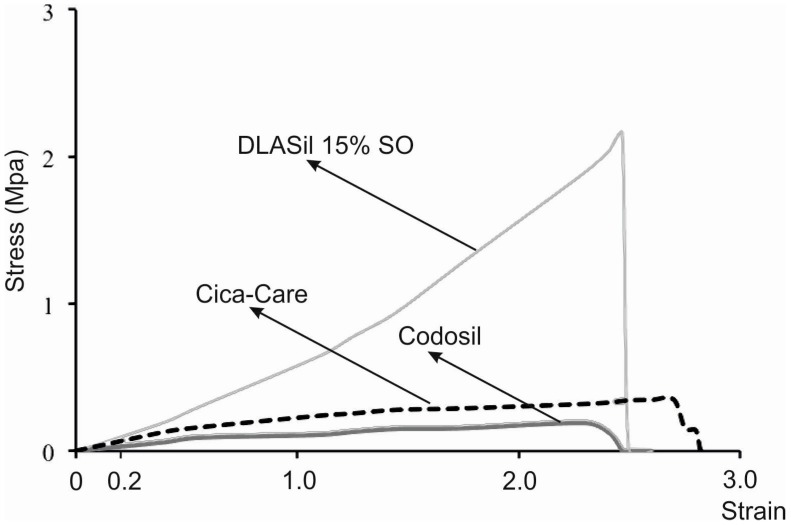
Comparison of the strain–stress curves obtained for Codosil™ and Cica-Care patches with Gumosil AD-1 film containing 15% *w*/*w* of silicon oil (SO).

Mechanical properties of silicone DLASil were compared with commercially available silicone patches, Codosil™ and Cica-Care, which were considered as applicable and possessing desirable elastic features for medical purposes (Table 1, Figure 5). Liquid polydimethylsiloxane, SO (kinetic viscosity 350 cSt), was chosen as an additive, which can be used as a levigating agent, substantial for obtaining a homogenous distribution of the active substances incorporated into the transdermal patch. Matrices formed with PDMS are highly hydrophobic with a relatively high density in comparison to other commonly used polymeric matrix-formers [18]. When excipients are added, significant changes in mechanical properties of the matrices are always reported. In some cases, hydrophobic solids (e.g., fumed silica) are combined with PDMS, causing increased hardness and durability of the silicone matrix material with simultaneously decreased diffusivity and modifying its mechanical behaviour (e.g., elasticity) [18].

Elastic behaviour of the prepared silicone films was described by material deformation under stress condition of the assay. A typical plot of the strain–stress curve obtained for DLASil, Cica-Care, and Codosil™ patches is presented in Figure 5. The examined materials showed both elastic and plastic deformation regions on the curve. Linear slope of the initial part of the curve indicates that Hook’s law holds for the elastic deformation part of the curve, hence modulus E (Young’s modulus) was determined [15]. Linearity in the strain region (from 0 to 0.2) was noted for both DLASil formulations and commercially available patches (Cica-Care and Codosil™), thereby defining modulus E as ratio of tensile stress to strain, expressed as a fraction of original length (deformation over original length). Increasing SO content was found to cause significant loss in modulus E values, which corresponds to increasing elasticity of modified DLASil films (Table 1). Modulus E values for Codosil™ patch were comparable to those determined for DLASil films without SO, which indicates similar elastic features. On the contrary, elasticity established for the Cica-Care patch indicated significantly lower stiffness, although in comparison to formulations of DLASil, modification with increasing SO content allows achievement of the patch’s elasticity at a comparable level.

**Figure 6:**
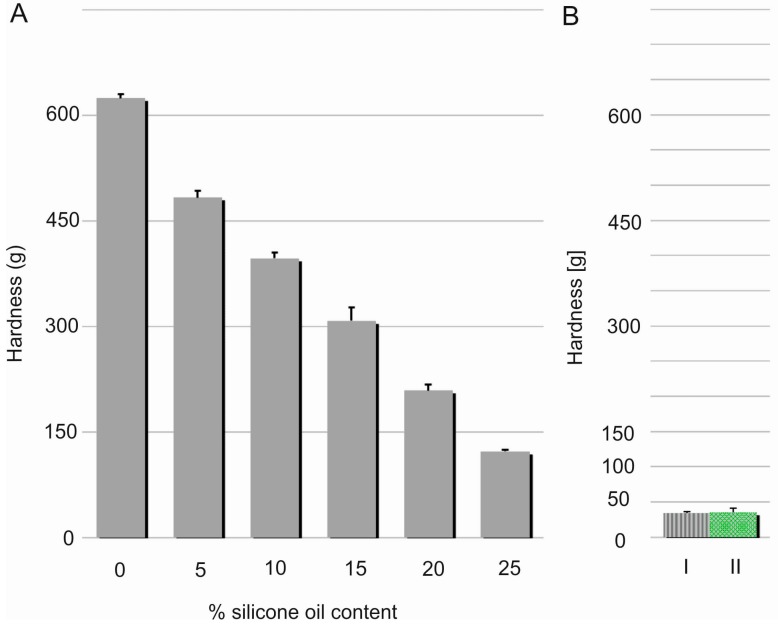
Correlation between hardness and silicone oil concentration in DLASil films (**A**). Hardness of Codosil™ (I) and Cica-Care (II) is presented additionally (**B**) (mean values ± standard deviation, *n* = 10).

Hardness, considered as material resistance to local deformation, is technically examined by penetration studies with a hard object [19]. Linear decrease (R^2^ = 0.9933) in the films’ hardness was noted with increasing content of the silicone oil (Figure 6). This effect can be probably explained by relaxation of a dense chain structure which is plasticised with SO. This assumption seems to be proven by simultaneous elasticity improvement expressed by modulus E values. Nevertheless, hardness of DLASil films containing 25% *w*/*w* of SO was found to be significantly higher in comparison to Codosil™ and Cica-Care patches. Though, it was still considered as approvable in terms of applicability of the newly formulated matrices. On the other hand, higher durability of DLASil allows its application without an additional supporting layer, while a very soft Codosil™ requires a backing layer.

Tensile strength (TS), which corresponds to material ability to withstand stress in tension mode, was calculated by dividing maximum force (N detected at break point by the cross-section area (mm^2^) of the sample [20]. The results are presented in Table 1. Under the same stress conditions, both Codosil™ and Cica-Care patches were proven to be considerably more fragile than DLASil film, which confirms the observation of the structures using SEM. Although durability of DLASil films constantly decreased as a function of increasing SO content in the matrix, even an additional 25% of the liquid component did not result in the same low tensile strength. Due to the oil sweating effect, it was impossible to further increase SO content.

Percentage elongation (%EB) was calculated by the equation *EB* = *∆l*/*l*_0_·100, where *∆l* is maximum elongation and *l*_0_ is initial length of sample [21]. The percentage elongation (%EB) parameter describes the capability of the material to resist permanent deformation. The effect of SO present in DLASil films was found to be insignificant, which indicates that basic polymeric structure—formed during the crosslinking reaction and responsible for extensibility of the films—appears to remain intact. Still, DLASil formulations were found in an acceptable range of endurance to withstand tensile strain, established on values noted for both commercially available products examined (Table 1).

Finally, mechanical characteristic of DLASil was found altered by SO addition into DLASil. In the case of the examined silicone elastomers, variation of tensile parameters of the films may be attributed to disturbance of the crosslinking reaction caused by the presence of silicone oil used as levigating agent (during incorporation of an active substance or a filler). Generally, the degree of crosslinking of the polymer depends on the length of the chain, molecular weight, and viscosity [17,22,23]. Therefore, insignificant reduction of the %EB value parameter suggests that the addition of SO does not affect the crosslinking reaction, so the basic polymeric DLASil structure is maintained. Considering flexibility and durability of SO-modified films, the observed alterations may be explained by the presence of free channels filled by silicone oil in submicroscopic structure of the polymers. Consequently, the relation between the compressive strength, the stiffness and the tensile strength will be affected [24].

Therefore, combined mechanical characterisation with morphology studies of polymer materials for medical use is beneficial, mostly for evaluation and optimisation during formulation of pharmaceutical product.

In the case of a dense polymer network, the lower ability to achieve maximum elongation can be seen, with a tendency to increase for DLASil, when the structure was relaxed by the addition of silicone oil. For properties such as tensile strength, elongation to break, and hardness of silicone elastomers, observed changes differ slightly. The possibility of adding silicone oil to the silicone matrix and assessing its optimum amount, which neither influences the crosslinking reaction nor the mechanical properties adversely, was evaluated. It should be emphasised that the addition of liquid components to silicone elastomers is not a standard practice. The study was planned to optimise the amount of silicone oil in the matrix, so as not to alter the mechanical properties (elongation, elasticity, and flexibility). It may happen that the relation between the compressive strength, the stiffness, and the tensile strength will change due to existing or emerging defects and cracks in the submicroscopic image of polymer structure [25]. Hence, it is important to study the morphology of polymer materials for medical use, since such observations may provide additional data to verify the mechanical properties of these materials.

### 3.3. Stability Studies

Further research was focused on investigation of the common environmental factors that may influence the stability of DLASil. When chemical stability of the formulation is taken into consideration, the effects of temperature, increased humidity, light, or oxidation—regarded as storage or stress conditions—are observed. The structure and morphology of silicone films were examined by means of optical and electron microscopy as well as spectroscopically by ATR–FTIR. Adhesiveness assessment was carried out to examine applicability maintenance of DLASil.

**Figure 7:**
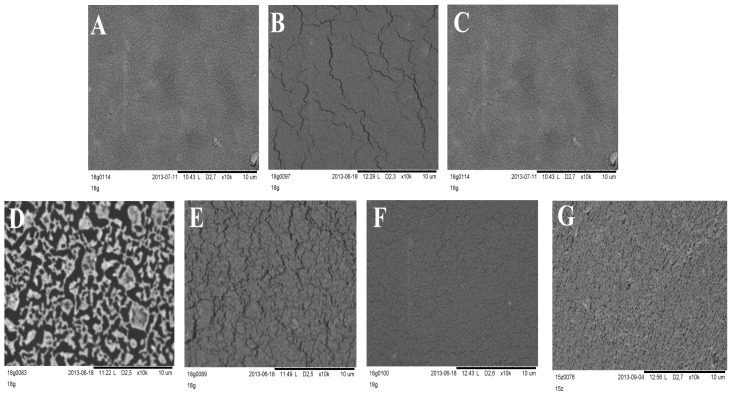
SEM images of DLASil layers’ surface: (**A**) reference—before testing; (**B**) stored for 28 days (40 °C, 75% RH); (**C**) refrigerated (4 °C, 28 days); (**D**) washed 10 times with a soap and water; (**E**) irradiated in the Suntest CPS+; (**F**) after mechanical test in a texture analyser; (**G**) Cica-Care without treatment.

Silicone films exposed to storage or stress conditions show no noticeable changes in their appearance. It was stated that the optical microscope technique was sufficient for general screening of morphology, but SEM allowed observation of detailed changes that occurred in layers during the stability test. SEM images presented in Figure 7 clearly show changes in the morphology of DLASil after exposition to test conditions. The most spectacular degradation in structure is observed for a sample which underwent 10-fold washing procedure. The pattern of the reference DLASil (Figure 7A) is presented as a smooth surface, however, this changed after washing the film: severe surface fragmentation indicates disturbed structure of silicone polymer (Figure 7D).

After irradiation of the DLASil samples in the Suntest chamber as well as after storage under stress conditions the change in the appearance of DLASil film in SEM images can be described as microcracking of the polymer structure.

**Figure 8:**
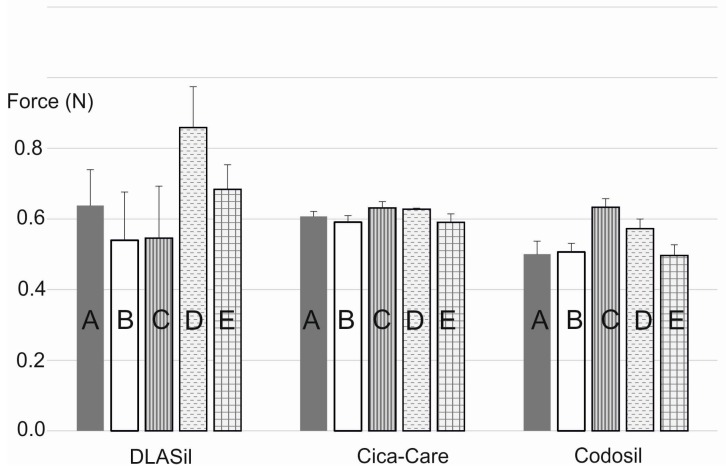
Adhesiveness of DLASil, Cica-Care, and Codosil exposed to storage conditions: (**A**) standard; (**B**) accelerated; (**C**) refrigeration; (**D**) UV exposure; (**E**) 10-fold wash (mean values ± standard deviation, *n* ≥ 10).

In Figure 8, the effect of storage and stress conditions on adhesiveness of the films is presented. Storage conditions (temperature and humidity) had no statistically significant effect on either DLASil or commercially available products. Only for Cica-Care and Codosil was a slight increase of the adhesive force noted after storage at 4–6 °C with increased humidity. The effect of stress factors such as UV light exposition and 10-fold wash, observed by SEM images analysis, was also confirmed by adhesion force measurement. The increase of adhesiveness was linked to the change of the adhesive layer surface and also in the structure of the silicone polymer, which occurred due to observed crack formation.

DLASil patches were designed for a maximum of 3–4 weeks of application; it was concluded that for the considered period, stability of the films was confirmed.

**Figure 9:**
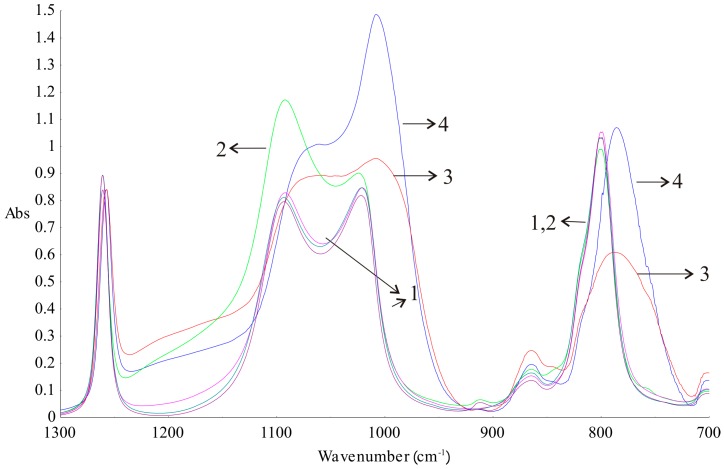
Attenuated total reflectance–Fourier-transformed infrared (ATR–FTIR) spectrum of silicone components (**1**—Gumosil Part B, DC A, DC B; **2**—Gumosil Part A) and silicone elastomers (**3**—Gumosil after curing reaction; **4**—DC after curing reaction).

Additionally, DLASil samples were characterised by means of ATR–FTIR spectroscopy after stability testing. The crosslinking process of silicone components depends on the presence of components with similar structures but possessing different functional groups at the ends of the molecule, as was shown by Pieńkowska, et al. [17]. When analysing the FTIR spectra (Figure 9), differences between silicone elastomers and primary components before crosslinking could be seen. The noticeable differences between the spectra are visible at 1092, 1021–1007, and 800–786 cm^−1^. This is due to the differences in the proportion of functional groups at the ends of the polymer chains before and after cure reaction. Bands signed as 3 and 4 in Figure 9 are identical, but they have lower intensities than the bands obtained for the silicone components before crosslinking. The clear separation of Si–O–Si bands at wavenumbers 1092 and 1021 cm^−1^ vanished as a consequence of crosslinking. The ATR–FTIR spectrum of the adhesive layer of DLASil has a higher intensity of bands compared to the bands of the nonadhesive elastomer. A sharp band at a wavenumber 1008 cm^−1^ can be a significant band, which indicates the characteristic arrangement of groups Si–O–Si and determines the adhesion properties of the elastomer [26].

**Figure 10:**
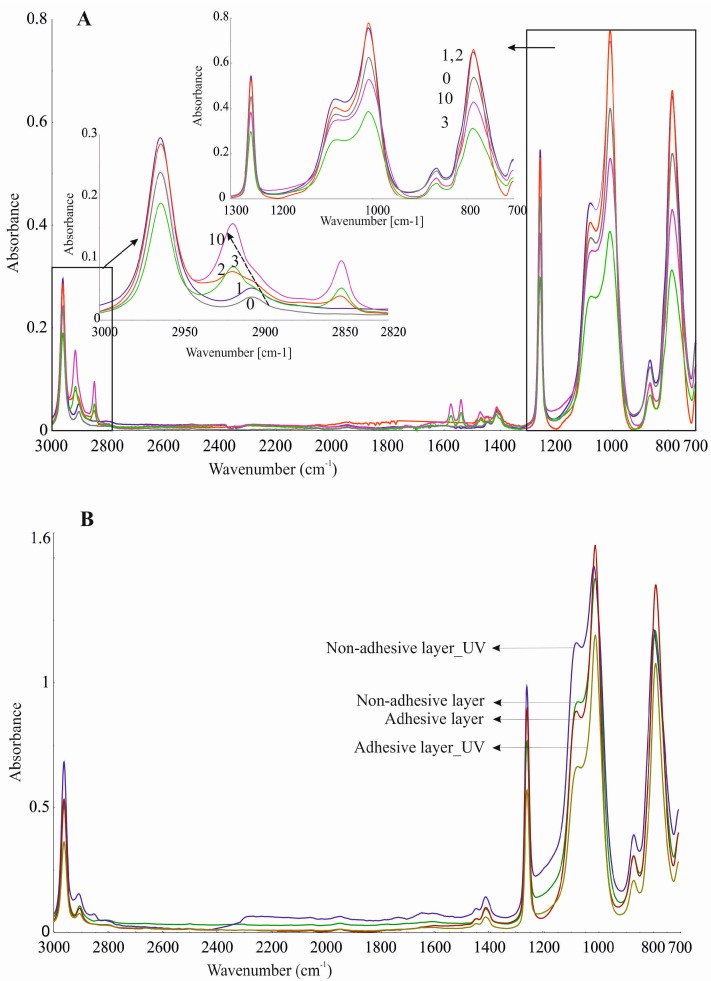
ATR–FTIR spectra recorded during stability testing of DLASil (**A**) adhesive layer during washing 0–10 times; (**B**) silicone elastomers after UV irradiation.

Silicone components used to form the silicone elastomer, DLASil, do not differ significantly in the infrared spectrum (Figure 9(1–2)). After curing, the characteristic bands of silicones are displaced and have little difference in shape, especially in the case of adhesive and nonadhesive layers’ spectrum. However, they are observed in the same region of wavenumbers in the infrared spectrum. ATR–FTIR spectra were also recorded after stability testing of each layer. Spectra presented in Figure 10A may indicate that the adhesive layer of DLASil is a little changed during washing. Bands in the spectra retained their shape and specific wavenumber. The only change in spectrum is observed in the intensity of bands. A clear increase of intensity is observed near wavenumber 2920 and 2850 cm^−1^. This region is responsible for asymmetrical and symmetrical valence (stretching) vibrations (C–H of the CH_3_ group). This situation may be caused by the residual soap after washing in the surface structure of the adhesive layer of DLASil. Interactions of the silicone patch with the soap during washing are so negligible that it retains the same appearance and unchanged chemical structure. Nevertheless, there are observed diverse and irregular changes of bands, but mostly due to higher intensity, not causing any wavenumber changes. Because an identical ATR–FTIR spectrum was achieved for stored DLASil at the higher temperature, its stability was confirmed.

Additional research performed on photostability testing of DLASil has shown that UV irradiation does not influence its structure. No difference in ATR–FTIR spectrum of the DLASil film before and after UV irradiation was observed (Figure 10B).

## 4. Conclusions

The selection of the silicone polymers in order to create the silicone adhesive dressing is related to the safety of their use, which is well documented, but also with the properties influenced by chemical structure. Any changes caused by the storage conditions of silicone films may influence the polymer structure, which can be observed by the morphology examination images or by mechanical properties determination. The presented relation between mechanical characterisation and morphology studies of the polymer materials for medical use was found to be beneficial, mostly for further evaluation and optimisation during development of pharmaceutical products.

Spatial arrangement of silicone groups in the polymer network, including, in particular, the bond angles and the type of groups directed outwards, is responsible for the adhesion to the surface, ergonomics, and durability of the medical material. The excipients incorporated into silicone matrix (e.g., silicone oil) may influence its physical or mechanical properties. Therefore, it is important to observe and study physical and chemical properties of the material intended for dermal use as dressings. Addition of SO is considered useful for adjustment of the required mechanical properties of the silicone patches, as was demonstrated in case of the effect on tensile parameters and elasticity of the nonadhesive layer of DLASil. Comparison between the examined DLASil and commercially available silicone patches indicated their similarity regarding elasticity, though Gumosil AD-1, even with high SO content (25%), was found to be more durable, which was considered beneficial for skin application purposes. Generally, DLASil demonstrated sufficient resistance to storage and stress factors, as shown by mechanical testing, and did not undergo structural damage, which was proved by ATR–FTIR analysis.

## Figures and Tables

**Table 1:** Tensile properties of examined silicone films: Gumosil AD-1 with different content of silicon oil (SO) and commercially available products (mean values ± standard deviation, *n* ≥ 8).

SO content *w*/*w* (%)	Tensile strength (MPa)	Elongation at break, EB (%)	Modulus E (MPa)
	Gumosil AD-1		
0	2.79 ± 0.53	310 ± 43	0.55 ± 0.03
5	2.47 ± 0.21	322 ± 14	0.49 ± 0.03
10	2.42 ± 0.33	302 ± 23	0.39 ± 0.03
15	2.34 ± 0.16	285 ± 17	0.35 ± 0.03
20	2.12 ± 0.38	283 ± 23	0.29 ± 0.01
25	2.02 ± 0.17	282 ± 18	0.27 ± 0.01
undefined	Codosil™		
	0.07 ± 0.01	212 ± 24	0.52 ± 0.08
undefined	Cica-Care		
	0.48 ± 0.05	345 ± 20	0.10 ± 0.01
